# The Clinicopathological and Prognostic Significance of SOX9 Expression in Gastric Cancer: Meta-Analysis and TCGA Analysis

**DOI:** 10.3389/fonc.2021.668946

**Published:** 2021-09-09

**Authors:** Guo Zu, Jiacheng Gao, Tingting Zhou

**Affiliations:** ^1^Department of Gastrointestinal Surgery, The Dalian Municipal Central Hospital Affiliated to Dalian Medical University, Dalian, China; ^2^Department of Graduate School, Dalian Medical University, Dalian, China; ^3^Department of Neurology, The First Affiliated Hospital of Dalian Medical University, Dalian, China

**Keywords:** SRY-box transcription factor 9 (SOX9), clinicopathological parameters, prognosis, gastric cancer, meta-analysis

## Abstract

**Background:**

The clinicopathological and prognostic significance of SRY-box transcription factor 9 (SOX9) expression in gastric cancer (GC) patients is still controversial. Our aim is to investigate the clinicopathological and prognostic value of SOX9 expression in GC patients.

**Methods:**

A systemic literature search and meta-analysis were used to evaluate the clinicopathological significance and overall survival (OS) of SOX9 expression in GC patients. The Cancer Genome Atlas (TCGA) dataset was used to investigate the relationship between SOX9 expression and OS of stomach adenocarcinoma (STAD) patients.

**Results:**

A total of 11 articles involving 3,060 GC patients were included. In GC patients, the SOX9 expression was not associated with age [odds ratio (OR) = 0.743, 95% CI = 0.507–1.089, p = 0.128], sex (OR = 0.794, 95% CI = 0.605–1.042, p = 0.097), differentiation (OR = 0.728, 95% CI = 0.475–1.115, p = 0.144), and lymph node metastasis (OR = 1.031, 95% CI = 0.793–1.340, p = 0.820). SOX9 expression was associated with depth of invasion (OR = 0.348, 95% CI = 0.247–0.489, p = 0.000) and TNM stage (OR = 0.428, 95% CI = 0.308–0.595, p = 0.000). The 1-year OS (OR = 1.507, 95% CI = 1.167–1.945, p = 0.002), 3-year OS (OR = 1.482, 95% CI = 1.189–1.847, p = 0.000), and 5-year OS (OR = 1.487, 95% CI = 1.187–1.862, p = 0.001) were significantly shorter in GC patients with high SOX9 expression. TCGA analysis showed that SOX9 was upregulated in STAD patients compared with that in normal patients (p < 0.001), and the OS of STAD patients with a high expression of SOX9 is poorer than that in patients with low expression of SOX9, but the statistical difference is not obvious (p = 0.31).

**Conclusion:**

SOX9 expression was associated with the depth of tumor invasion, TNM stage, and poor OS of GC patients. SOX9 may be a potential prognostic factor for GC patients but needs further study.

**Systematic Review Registration:**

PROSPERO, ID NUMBER 275712.

## Introduction

As the fifth most common malignant tumor, gastric cancer (GC) also is the third most frequent cause of cancer-related death worldwide (1). Almost half of these patients came from East Asia, including China (2). Although endoscopic examinations and treatment technology were performed to help in improving the diagnosis and treatments of GC, the prognosis of GC patients is still poor (3). Until now, TNM stage is still used as the major prognostic factor of GC clinically. However, even within the same TNM stage of tumor patients, there is a great difference in the aggressiveness of the tumor (4). Therefore, finding a prognostic biomarker to distinguish different tumor biological behaviors and prognosis of GC patients is needed.

As a transcription factor, SRY-box transcription factor 9 (SOX9) belongs to the SOX family and is involved in many physiological and pathological processes, such as cell growth, apoptosis, invasion, and metastasis of tumor cells (5, 6). Studies showed that SOX9 is highly expressed and could predict prognosis in many kinds of tumors, including hepatocellular carcinoma, colon cancer, and other cancer tissues (7, 8). There are also some reports about the role of SOX9 in GC patients. Mesquita et al. (9) reported that SOX9 expression was not related to the clinicopathologic characteristics but was a biomarker of relapse in GC patients. The prognostic significance of SOX9 expression in GC patients remains controversial. Choi et al. (10) found that SOX9 could not serve as a prognostic biomarker for GC patients. Richtig et al. (7) found that SOX9 high expression was a predictor of poor 5-year overall survival (OS) of GC. Until now, there is still no meta-analysis to investigate the relationship between SOX9 expression and clinicopathological and prognostic value of GC. Therefore, we performed a meta-analysis and The Cancer Genome Atlas (TCGA) analysis to investigate the clinicopathological and prognostic significance of SOX9 expression in GC patients.

## Methods

### Search Strategy

We searched the following electronic databases: Web of Science, PubMed, Cochrane Library, and China databases (CNKI and CBM) from the date of establishment until December 31, 2020. The following terms were used to search: “SOX9” or “SRY related high-mobility group box 9” and “gastric” or “stomach” and “carcinoma” or “cancer”.

### Inclusion and Exclusion Criteria

The inclusion criteria were used for screening the articles: 1) To evaluate the association between SOX9 expression and clinicopathological and/or prognostic significance in GC patients, 2) SOX9 expression in GC tissue was measured by immunohistochemistry (IHC).

The following exclusion criteria were included in this meta-analysis: 1) The article included same population or data, 2) The studies only included cell models, 3) lack of clinical data and statistical analysis.

### Data Extraction and Study Assessment

Two authors (GZ and JG) searched and screened the manuscripts according to the inclusion criteria independently. A third author (TZ) discussed and resolved any discontent. The following data were extracted: first author, year of publication, number of cases, stage of tumor invasion, lymph node metastasis, TNM stage, OS of GC patients, and SOX9 expression. The Newcastle–Ottawa Scale (NOS), which included patient selection, comparability, and outcome, was used to assess the quality of the manuscript. NOS score higher than 6 was considered high quality and included in our meta-analysis.

### Analysis of The Cancer Genome Atlas Datasets

A dataset including information on RNAseqV2 and clinical data of stomach adenocarcinoma (STAD) patients was obtained from TCGA datasets (https://genome-cancer.soe.ucsc.edu). GEPIA2 was used to analyze RNA sequencing (RNA-seq) data. SOX9 expression analysis in STAD and normal gastric tissues was conducted using one-way ANOVA. Kaplan–Meier method and log-rank test were used for survival analysis.

### Statistical Analysis

All analyses were performed with Stata 10.0 software. Q-test and I^2^ index were used to assess the heterogeneity of included studies. The 95% confidence intervals (CIs) of mean differences were calculated using a fixed-effects model; when I^2^ > 50%, a random-effects model was performed. Pooled odds ratio (OR) with a 95% CI was calculated to investigate the relationship between SOX9 expression and clinicopathological and prognostic parameters. Funnel plots were used to evaluate the publication bias. p–values ≤0.05 were considered a significant difference.

## Results

### Description of Studies

**Figure 1:**
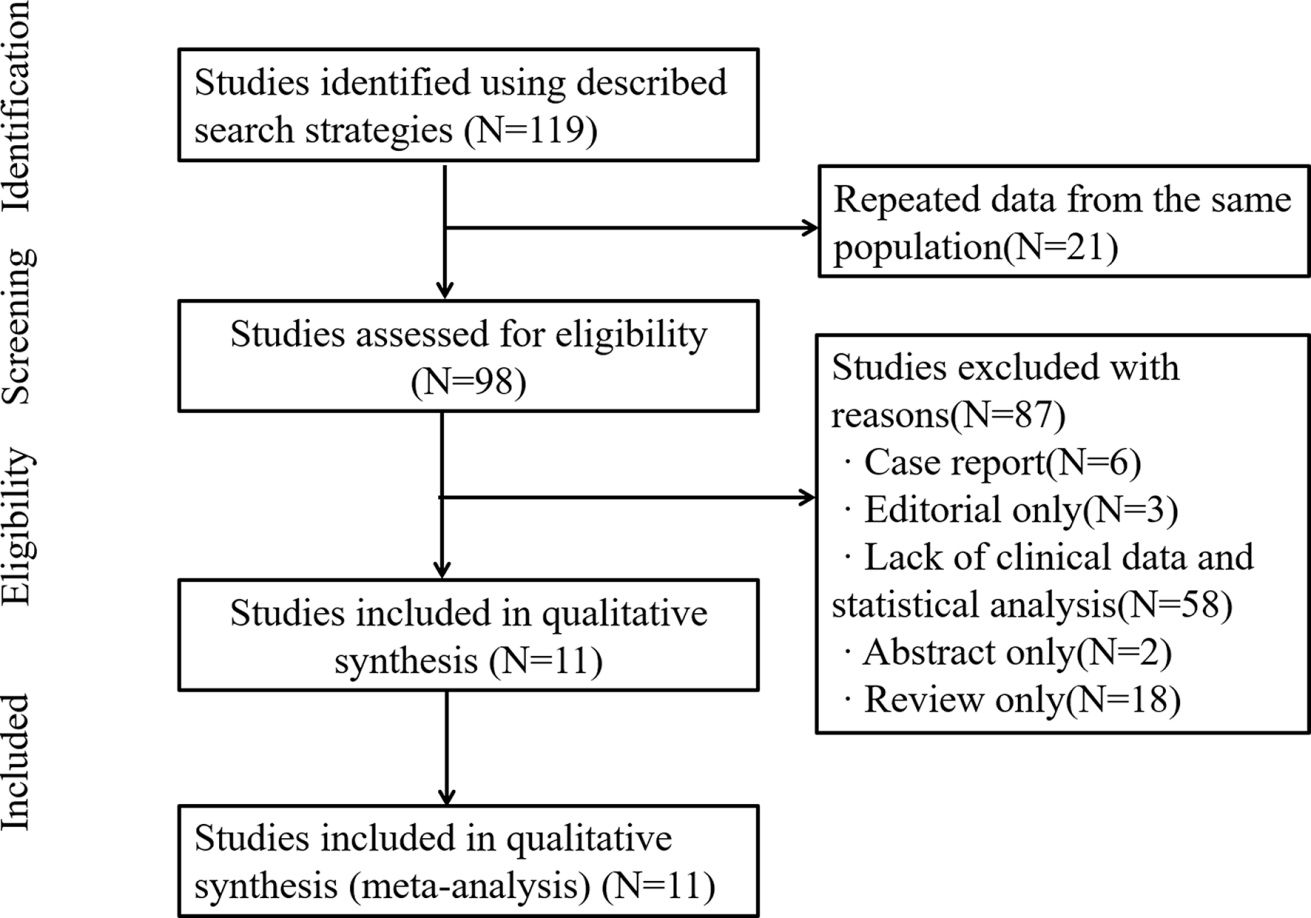
Flowchart for selection of studies.

Our study identified 119 articles in databases. Among them, 21 articles were excluded due to repeated data. In addition, 87 articles were excluded due to lack of clinical data and statistical analysis, case report and review. Finally, 11 relevant articles and 3,060 GC patients were included for quantitative analysis in this meta-analysis (7, 9–18) (**Figure 1**). The results of extracted data in our included studies were listed in **Table 1**.

**Table 1:** Main characteristics and results of the eligible studies.

No.	First author	Year	No.	Gender (M/F)	pT stage (T1–2/T3–4)	pN stage (N+/N-)	During	Country	Method	NOS
1	Choi [10]	2014	185	–	17/168	181/4	–	Japan	IHC	8
2	Shao [11]	2012	112	80/32	22/90	79/33	2004–2006	China	IHC	7
3	Zhou [12]	2010	186	–	86/100	152/34	2002–2006	China	IHC	8
4	Mesquita [9]	2019	333	188/145	–	–	2008.1–2014.12	Portugal	IHC	8
5	Zhang [13]	2018	195	76/26	27/77	70/23	2014.3–2055.4	China	IHC	6
6	Link [14]	2018	211	135/78	102/109	194/17	2002–2014	Germany	IHC	6
7	Sun [15]	2012	382	274/108	–	157/225	1990–2008	Japan	IHC	7
8	Richtig [7]	2017	876	–	–	–	–	Austria	IHC	7
9	Liu [16]	2011	417	–	70/85	131/24	2005–2008	China	IHC	6
10	Liu [17]	2017	50	36/14	24/26	22/28	2009–2012	China	IHC	6
11	Lv [18]	2014	113	67/46	–	50/63	2010.1–2012.12	China	IHC	6

IHC, immunohistochemistry; -, not reported; M, male; F, female; NOS, Newcastle–Ottawa Scale.

### SOX9 Expression and Clinicopathological Parameters

**Figure 2:**
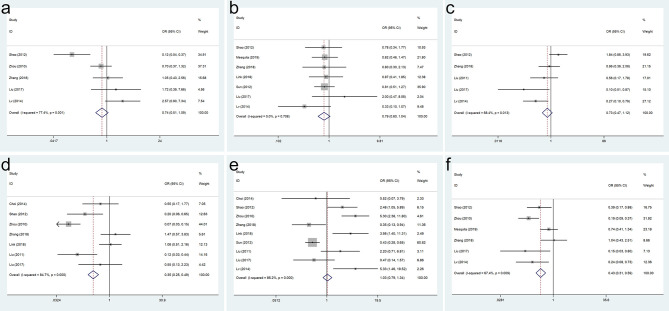
Forest plots for the association of SRY-box transcription factor 9 (SOX9) expression with clinicopathological parameters: **(A)** age, **(B)** sex, **(C)** differentiation, **(D)** depth of invasion, **(E)** lymph node metastasis, and **(F)** TNM stage.

As shown in **Figure 2**, the meta-analysis results of included studies showed that SOX9 expression was not associated with age (OR = 0.743, 95% CI = 0.507–1.089, p = 0.128), sex (OR = 0.794, 95% CI = 0.605–1.042, p = 0.097), differentiation (OR = 0.728, 95% CI = 0.475–1.115, p = 0.144), and lymph node metastasis (OR = 1.031, 95% CI = 0.793–1.340, p = 0.820) in GC patients. Our analysis indicated that SOX9 expression was associated with depth of invasion (OR = 0.348, 95% CI = 0.247–0.489, p = 0.000) and TNM stage (OR = 0.428, 95% CI = 0.308–0.595, p = 0.000) in GC patients (**Figure 2**).

**Figure 3:**
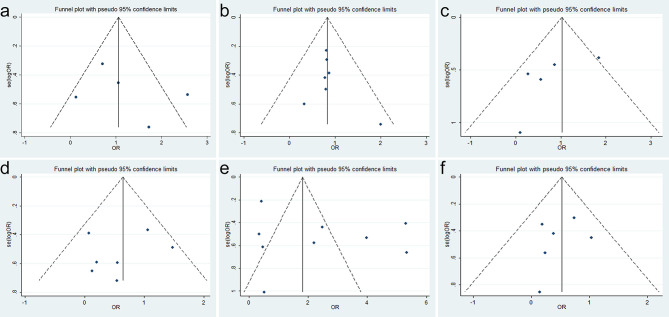
Funnel plots for SRY-box transcription factor 9 (SOX9) expression with clinicopathological parameters: **(A)** age, **(B)** sex, **(C)** differentiation, **(D)** depth of invasion, **(E)** lymph node metastasis, and **(F)** TNM stage.

No significant publication bias was confirmed to exist in age, sex, differentiation, depth of tumor invasion, lymph node metastasis, and TNM stage because their p-values were larger than 0.05 in Egger’s test (p = 0.858, p = 0.991, p = 0.082, p = 0.917, p = 0.196, and p = 0.482, respectively) (**Figure 3**).

### Correlation of SOX9 Expression With Overall Survival

**Figure 4:**
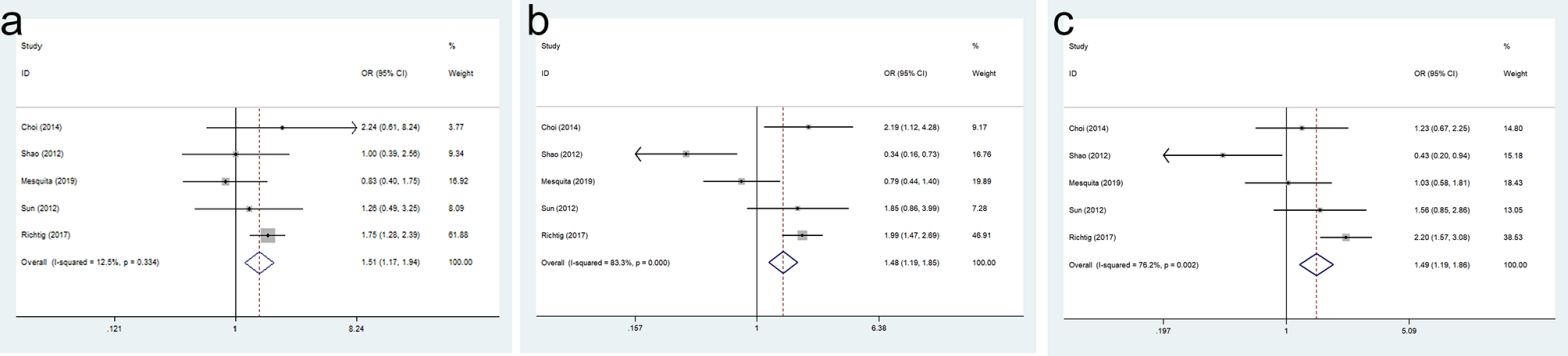
Forest plot for the association of SRY-box transcription factor 9 (SOX9) expression with overall survival (OS) of gastric cancer (GC) patients: **(A)** 1-year OS, **(B)** 3-year OS, and **(C)** 5-year OS.

The result of SOX9 expression in the OS of GC patients was shown in **Figure 4**. Compared with GC patients with a low SOX9 expression, the 1-year OS (OR = 1.507, 95% CI = 1.167–1.945, p = 0.002), 3-year OS (OR = 1.482, 95% CI = 1.189–1.847, p = 0.000), and 5-year OS (OR = 1.487, 95% CI = 1.187–1.862, p = 0.001) were significantly shorter in GC patients with high SOX9 expression.

### Publication Bias Between SOX9 Expression and Overall Survival

**Figure 5:**
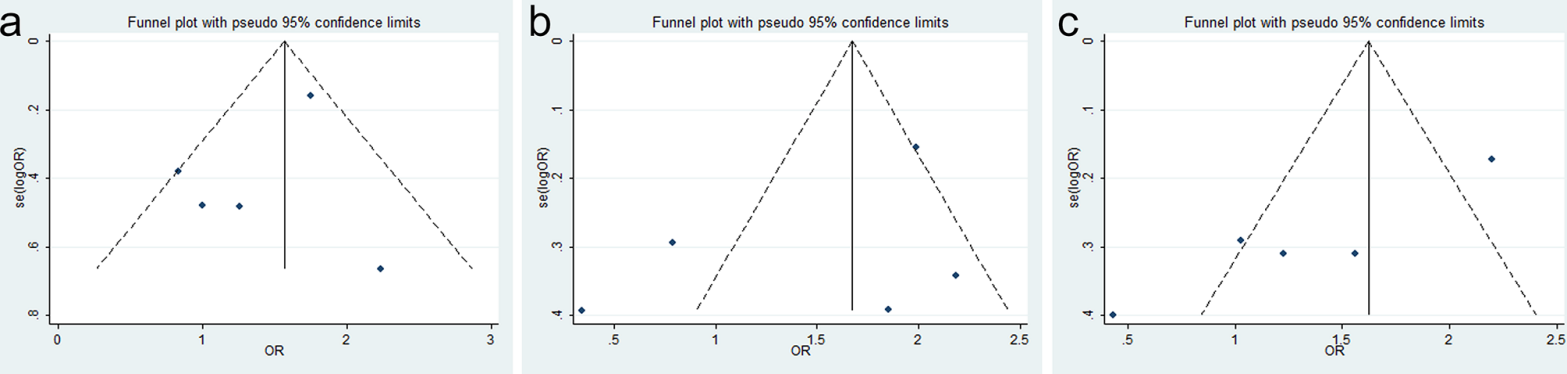
Funnel plots for SRY-box transcription factor 9 (SOX9) expression with overall survival (OS) of gastric cancer (GC) patients: **(A)** 1-year OS, **(B)** 3-year OS, and **(C)** 5-year OS.

There was also no evidence for obvious publication bias in 1-, 3-, and 5-year OS (Egger’s test, p = 0.356, p = 0.323, and p = 0.462, respectively) (**Figure 5**). The finding was another strong evidence to verify that SOX9 was a prognostic factor for GC patients.

### The Cancer Genome Atlas Analysis

**Figure 6:**
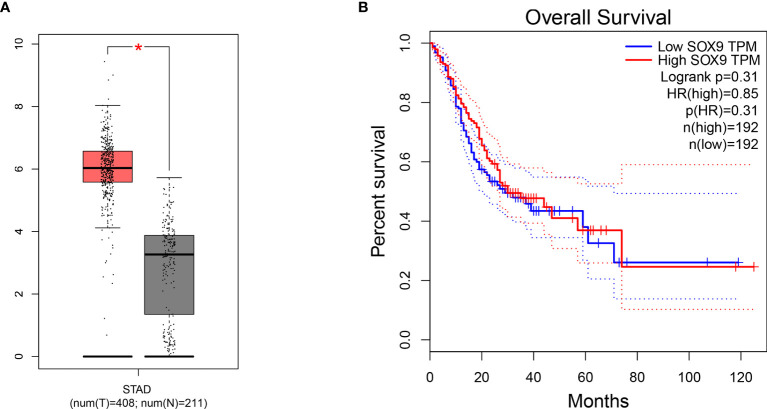
The relationship between SRY-box transcription factor 9 (SOX9) expression and prognostic value in gastric cancer (GC) patients in The Cancer Genome Atlas (TCGA) cohort. **(A)** The expression level of SOX9 in GC tissue and normal gastric tissue (p < 0.001). T, GC tissue; N, normal gastric tissue. **(B)** Overall survival (OS) plots of SOX9 in GC patients in TCGA cohort (log-rank p = 0.31). * means that P < 0.05 versus T group.

To further investigate the relationship between SOX9 expression and prognostic value in GC patients, TCGA clinical data were used. The dataset included 408 STAD and 211 normal gastric controls. The result showed that SOX9 expression was upregulated in STAD patients (|Log2FC| Cutoff >1, q-value <0.01, p < 0.001) (**Figure 6A**). Furthermore, 384 STAD patients were divided into SOX9 high-expression group (n = 192) and SOX9 low-expression group (n = 192). The OS of STAD patients with SOX9 high expression was poorer than that of the patients with low-expression SOX9, but there was no statistical difference (p = 0.31) (**Figure 6B**).

## Discussion

Previous studies have investigated the correlation between transcription factors and carcinogenesis of various cancers, including GC (19, 20). As a transcriptional factor, SOX9 was significantly correlated with tumor invasion and metastasis of GC cells and might promote gastric tumor progression (12, 16). However, Choi et al. (10) find that there is no relationship between SOX9 and poor differentiation, lymph node metastasis, tumor invasion, and tumor site of GC, and SOX9 was not a prognostic biomarker for patients with GC (10). Additionally, other researchers revealed that the expression of SOX9 in GC tissues was significantly higher compared with adjacent normal tissues; however, SOX9 was not related to tumor size, lymph node metastasis, distant metastasis, and TNM staging (13). In our results of meta-analysis, we found that 1) SOX9 was associated with the depth of invasion and TNM stage of GC; 2) SOX9 was not associated with age, sex, differentiation, and lymph node metastasis of GC patients; 3) SOX9 might be a potential prognostic factor for GC patients.

It has been reported that SOX9 expression might promote tumor progression *via* activating proliferation and metastasis (21, 22). SOX9 was overexpressed in advanced GC and related to the progression of the tumors and depth of tumor invasion. Moreover, SOX9 expression was elevated markedly in the progression of lymph node metastasis and tumor staging. Sun et al. (15) found that SOX9 was associated with tumor location, clinical stage, lymph node metastasis, venous infiltration, and nodal metastasis. The result is not consistent with that of other studies (13). In our study, due to lack of enough data on tumor size and distant metastasis, we could not analyze the association between SOX9 expression and tumor size and distant metastasis. We just analyzed the association between SOX9 expression and depth of invasion, lymph node metastasis, and TNM stage. Our results showed that SOX9 expression was associated with the depth of invasion and TNM stage of GC patients but not associated with lymph node metastasis, age, gender, and tumor differentiation. This may be due to the small sample or lack of data on tumor size and distant metastasis. It is necessary to analyze the relationship between SOX9 and tumor size and distant metastasis in a larger sample of GC.

Several studies have revealed the relationship between SOX9 expression and prognosis of tumor patients. Osman et al. (23) reported that SOX9 positive expression had significantly shorter OS in hepatocellular carcinoma patients. Tang et al. (24) found that SOX9 promoted cell proliferation, migration, and stemness and predicted poor prognosis of triple-negative breast cancer patients. The association between SOX9 expression and prognosis of GC patients has been investigated, but the role of SOX9 expression and prognosis of GC patients is controversial. Choi et al. (10) found that SOX9 could not serve as a prognostic biomarker for GC patients. Sun et al. (15) reported that SOX9 was not associated with prognosis of GC patients. However, Richtig et al. (7) found that high SOX9 expression prevailed as a strong predictor of poor OS of GC. Our meta-analysis results found that the OS of GC patients with high SOX9 expression was significantly shorter compared with that of GC patients with low SOX9 expression. The results of bioinformatic analysis confirmed that SOX9 expression is upregulated in STAD patients compared with normal patients, and the OS of patients with a high expression of SOX9 was poorer than that of patients with a low expression of SOX9, but there was no significant statistical difference. This may be due to the small sample or pathological type of data, and the bioinformatic analysis samples that only include 384 patients with STAD, but not all pathological types of GC patients. Therefore, the effect of SOX9 expression on the OS of GC patients needs further study that includes more articles and patients.

Several limitations of our meta-analysis should be acknowledged. First, the included studies were only published in English and Chinese, so the introduced bias was not neglected. Second, the results are subjectively assessed by examiners, and the data of prognosis and expressions of SOX9 in GC tissue were all tested by IHC. The extracted cutoffs (+/- or high/low) on SOX9 expression were not all the same in GC tissues, and standards of SOX9 expression were subjective. This may affect the heterogeneity or bias of our results. Furthermore, we investigated SOX9 expression and the clinicopathological parameters and prognosis of GC patients and performed with Stata software for binary data. It is much better to analyze more factors (including age, gender, depth of tumor invasion, TNM stage, and SOX9 expression) of OS by multivariate analysis to further confirm the conclusion. In addition, it could be much better to further investigate the association between SOX9 and the molecular subtypes defined in TCGA [p53-, p53+, microsatellite instability (MSI), epithelial–mesenchymal transition (EMT)] or EMT subtype (25). We will explore the relationship between SOX9 and the molecular subtypes or EMT subtype according to the inclusion of the second most important CG cohort [Asian Cancer Research Group (ACRG)] in the future.

## Conclusion

We performed a meta-analysis to investigate SOX9 expression and the clinicopathological and prognostic significance of GC patients. Our results showed that SOX9 could influence depth of invasion, TNM stage, and poor OS of GC patients but not associated with age, sex, differentiation, and lymph node metastasis. SOX9 might a potential prognostic factor for GC patients, but more studies are needed to confirm the value of the current meta-analysis.

## Data Availability Statement

The original contributions presented in the study are included in the article/**Supplementary Material**. Further inquiries can be directed to the corresponding author.

## Author Contributions

Conceived and designed the experiments: GZ. Performed the experiments: GZ, JG, and TZ. Analyzed the data: GZ and JG. Contributed reagents/materials/analysis tools: GZ, JG, and TZ. Wrote the paper: GZ and JG. All authors contributed to the article and approved the submitted version.

## Funding

This work was supported by grants from the National Natural Science Foundation of China (grant number 81700465), the Natural Science Foundation of Liaoning Province, China (No. 2019-BS-059), and the High-level Talents Innovation Plan of Dalian, China (No. 2018RQ27).

## Conflict of Interest

The authors declare that the research was conducted in the absence of any commercial or financial relationships that could be construed as a potential conflict of interest.

## Publisher’s Note

All claims expressed in this article are solely those of the authors and do not necessarily represent those of their affiliated organizations, or those of the publisher, the editors and the reviewers. Any product that may be evaluated in this article, or claim that may be made by its manufacturer, is not guaranteed or endorsed by the publisher.
