# Supplementary material for: The Clinicopathological and Prognostic Significance of SOX9 Expression in Gastric Cancer: Meta-Analysis and TCGA Analysis
Source: Front Oncol. 2021 Sep 9;11:668946. doi: 10.3389/fonc.2021.668946 (PMC8458960; doi:10.3389/fonc.2021.668946)
Supplement: Supplementary file 1 [file DataSheet_1.docx]

**supplementary data**

**Expression of SOX9 and subtype of gastric cancer**

A total of 1105 patients in six studies were included to detect the relationship between subtype of gastric cancer (intestinal type and diffuse type) and expression of SOX9 in this meta-analysis. We found an association between subtype of gastric cancer and expression of SOX9, and OS of patients with intestinal type is much better than diffuse type patients (OR: 1.810, 95%CI: 1.391-2.356, P = 0.000) (Supplementary Fig. 1).


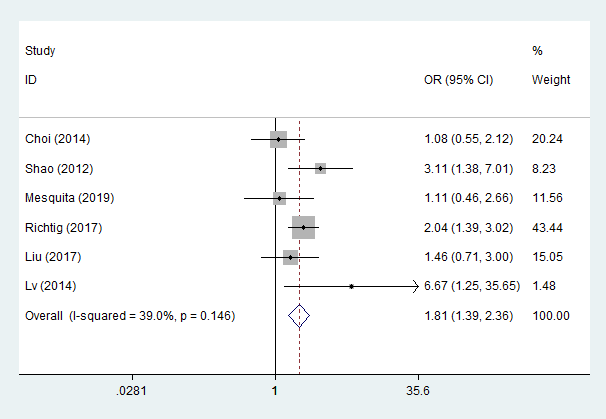

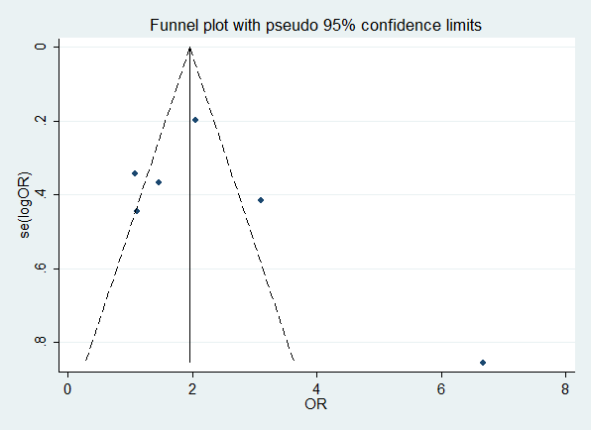


Supplementary Fig. 1 Forest plots showed that the association between SOX9 expression and subtype of gastric cancer (intestinal type and diffuse type). a: forest plots, b: funnel plots.
